# Numerical Simulation and Experimental Validation of the Resistance of Recycled Aggregate Concrete to Chloride Penetration Under Freeze-Thaw Cycles

**DOI:** 10.3390/ma19153342

**Published:** 2026-08-06

**Authors:** Yuze Li, Jiayi Zhao, Qifeng Liu, Xiaoyang Chen, Haiwei Zhang, Kairong Jin, Wei Wang, Peng Yin, Tingting Zhang

**Affiliations:** 1 School of Materials and Metallurgy, University of Science and Technology Liaoning, Anshan 114051, China; lyz050705@126.com (Y.L.); lqf5756960@163.com (Q.L.); 2 School of Civil and Hydraulic Engineering, Ningxia University, Yinchuan 750021, China; 3 State Key Laboratory of Water Engineering Ecology and Environment in Arid Area, Inner Mongolia Agricultural University, Hohhot 010018, China; 4 Ningxia Construction Project Quality and Safety General Station, Yinchuan 750021, China; 5 School of Civil Engineering, Dalian University of Technology, Dalian 116024, China

**Keywords:** recycled aggregate concrete, chloride penetration, freeze-thaw cycles, numerical simulation, experimental validation

## Abstract

This study investigated the resistance of recycled aggregate concrete (RAC) to chloride penetration under freeze-thaw cycles using both numerical simulation and experimental validation. A five-phase mesoscale model of RAC was developed. The salt freeze-thaw test was conducted to validate the simulated values and explore the effects of different recycled coarse aggregate (RCA) substitution ratios, as well as varying dosages of fly ash and superfine fly ash, on chloride concentration in RAC. The experimental data showed satisfactory agreement with the simulated values, confirming the model’s validity. Additionally, the RCA volume fraction, interfacial transition zone (ITZ) thickness, and adhesive ratio of old mortar were analyzed using simulation. The variation patterns of compressive strength, relative dynamic elastic modulus, and mass loss of RAC also revealed the mechanisms by which RCA substitution ratio and the dosages of fly ash and superfine fly ash act under salt freeze-thaw conditions. This study provides guidance for enhancing the resistance of RAC to chloride penetration and its durability under freeze-thaw conditions.

## 1. Introduction

In recent years, the urbanization process has led to a significant increase in construction and demolition waste (C&DW). Finding ways to reuse C&DW has become an urgent issue that needs to be addressed [1,2,3]. Recycled concrete technology maximizes the potential of C&DW by using it as recycled aggregates, providing partial or complete substitutes for natural aggregates in pouring new concrete [4,5]. Recycled aggregates contain more pores and microcracks, which accelerate chloride penetration and impair the service life of buildings [6,7,8]. Some scholars have investigated the resistance of concrete to chloride penetration by treating concrete as a multi-phase inhomogeneous material at the mesoscale [9,10]. Xiao et al. [11] proposed a five-phase composite model with one RCA and studied chloride migration in the model using numerical analysis. Ying et al. [12] established a five-phase model with multiple spherical structures for RAC, considering the adhesive old mortar and ITZ. Strubar III [13] proposed a one-dimensional chloride permeation modeling of RAC as a composite material composed of five phases to investigate its service life. Liu et al. [14] analyzed the impact of individual concrete components on chloride penetration using a three-phase modeling approach to simulate multiple-component ion transfer. Hu et al. [15] established a stochastic five-phase model for RAC to quantitatively analyze each phase’s impact upon RAC’s effective chloride spreading rate. Yu and Lin [16] defined RAC as a three-phased composite and investigated the effects of key parameters on chloride diffusion. Additionally, an approach based on genetic programming was proposed to forecast the permeability of RAC. Wu et al. [17] developed a convex polygon computational model by considering RAC as a seven-phase composite structure containing cracks and damage zones. In summary, most relevant studies adopt Fick’s second law as the core governing equation. Researchers have established multiphase inhomogeneous models to reconstruct the mesostructural features of RAC, which can effectively characterize internal chloride diffusion behavior. Nevertheless, the majority of these numerical studies lack adequate experimental validation. Furthermore, they ignore the coupled effects of freeze-thaw cycles and supplementary cementitious materials on chloride diffusion, limiting their reliability for predicting long-term concrete durability under actual engineering service environments.

Concrete structures in cold regions are susceptible to freeze-thaw damage. During freeze-thaw cycles, the freezing of pore water generates hydraulic and crystallization pressures, which induce microcracking and cumulative deterioration in concrete. It is crucial to analyze the characteristics of chloride penetration in concrete exposed to freeze-thaw cycles [18,19,20,21,22]. Kuosa et al. [23] conducted field and laboratory tests to investigate the effects of freeze-thaw and chloride migration on concrete durability. The results indicated that freeze-thaw-induced microcracks significantly accelerate chloride ingress in concrete. Jiang et al. [24] proposed a three-phase mesoscale method by introducing time-varying porosity to study chloride diffusion under freeze-thaw conditions. Qiang et al. [25] calculated the total charge to explore the variation in concrete chloride content during freeze-thaw cycles with partial cement substitution by fly ash or silica fume. Yin et al. [26] conducted pull-out and flexural tests to evaluate the performance of textile-reinforced concrete under freeze-thaw damage. The study found that the mechanical properties decreased significantly with the increase of freeze-thaw cycles, especially after 90 cycles. Gao et al. [27] established a multi-phase chloride diffusion model for concrete under freeze-thaw cycles by considering the evolution of pore structure and cracks, and validated the model with third-party test data. Wang et al. [28] carried out chloride transport tests considering the combined effects of dry-wet ratio and freeze-thaw cycles. The experimental results showed that the relative error between measured data and the proposed empirical prediction values of chloride concentration was less than 15%. Existing studies have achieved fruitful outcomes, yet systematic research combining experimental testing and numerical simulation to explore the chloride diffusion behavior of RAC under freeze-thaw cycles remains scarce. Therefore, it is necessary to conduct both experimental and numerical investigations to analyze the chloride diffusion process of RAC under freeze-thaw conditions.

In previous studies, many existing chloride penetration models for RAC adopted third-party experimental data to validate the simulated results. In this study, a five-phase mesoscale model was developed by setting a specific diameter for coarse aggregate. The single-side salt freeze-thaw test was employed to validate the simulated values. This study aims to investigate the effects of different RCA substitution ratios, as well as varying dosages of fly ash and superfine fly ash, on chloride concentration in RAC. Additionally, the effects of the key model components on chloride concentration were further explored.

## 2. The Basics of Numerical Simulation

### 2.1. Chloride Migration Theory

When the RAC is wholly saturated, the chloride migration is solely determined by diffusion. Fick’s second law states that the behavior of chloride diffusion can be described by Equation (1):

(1) $$\frac{\partial C_{s}\left(x_{0},y_{0},t_{0}\right)}{\partial t_{0}}=D_{p}\left(\frac{\partial C_{s}\left(x_{0},y_{0},t_{0}\right)}{\partial {x_{0}}^{2}}+\frac{\partial C_{s}\left(x_{0},y_{0},t_{0}\right)}{\partial {y_{0}}^{2}}\right)(p=1,2,3,4,5)$$

where $t_{0}$ is the time of chloride penetration (s), $x_{0}$ is the depth of chloride penetration (m), $C_{s}(x_{0},y_{0},t_{0}$) is the chloride concentration at time $t_{0}$ and position $\left(x_{0},y_{0}\right)$ (%), $D_{p}$ is the chloride diffusion coefficient of phase p (new mortar, old mortar, new ITZ, old ITZ, and original aggregate) (m^2^/s).

To improve the convenience of model analysis, two specific conditions were defined with the following equations:

(2) $$C_{s}\left(x_{0},y_{0},0\right)=0$$

(3) $$C_{s}\left(0,y_{0},t_{0}\right)=C_{t}$$

where $C_{t}$ is the chloride concentration at the model’s boundary (%).

### 2.2. Recycled Aggregate Gradation

Walraven presented the area distribution of coarse aggregate on a two-dimensional plane based on Fuller’s three-dimensional aggregate grading curve [29]. This can be calculated using Equation (4):

(4) $$P_{m}\left(D<D_{c}\right)=P_{s}\left(1.065{D_{c}}^{0.5}{D_{max}}^{-0.5}-0.053{D_{c}}^{4}{D_{max}}^{-4}\;-0.012{D_{c}}^{6}{D_{max}}^{-6}-0.045{D_{c}}^{8}\right.{D_{max}}^{-8}+0.025{D_{c}}^{10}\left.{D_{max}}^{-10}\right)$$

where $P_{m}$ is the possibility of the coarse aggregate diameter in $D<D_{c}$ range, $P_{s}$ is the volume fraction of coarse aggregate, $D_{c}$ is the coarse aggregate diameter (mm), $D_{max}$ is the maximum diameter of coarse aggregate (mm).

The area of coarse aggregate in different diameter ranges was determined using Equation (5):

(5) $$S_{c}\left[D_{i},\;D_{i+1}\right]=\frac{P_{m}\left(D_{i+1}\right)-P_{m}\left(D_{i}\right)}{P_{m}\left(D_{max}\right)-P_{m}\left(D_{min}\right)}\times S_{b}\times S_{a}$$

where $S_{c}$ is the area of coarse aggregate in $\left[D_{i},\;D_{i+1}\right]$ diameter range (mm^2^), $S_{b}$ is the proportion of coarse aggregate area, $S_{a}$ is the model area (mm^2^), $D_{min}$ is the minimum diameter of coarse aggregate (mm).

### 2.3. Five-Phase Mesoscale Model of RAC

**Figure 1:**
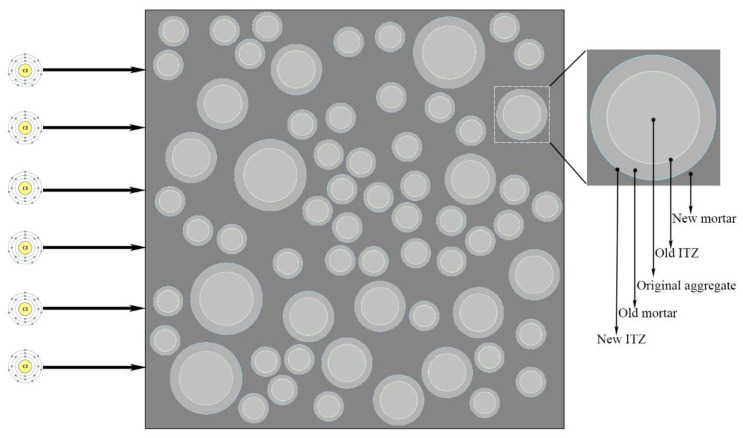
Schematic diagram of five-phase mesoscale model configured with 100% RCA.

The five-phase mesoscale model configured with 100% RCA was developed using MATLAB R2020a and subsequently imported into COMSOL Multiphysics 5.6, as shown in Figure 1. It comprised new mortar, adhesive old mortar, new ITZ, old ITZ, and original aggregate, with dimensions of 100 mm × 100 mm. The coarse aggregate diameters ranged from 5 mm to 20 mm. To simplify the modeling process, three specific diameters of coarse aggregate (7 mm, 12 mm, and 17 mm) were selected. The left boundary of the model was designated as the region for chloride penetration.

### 2.4. Parameter Setting

RAC is an inhomogeneous material with different chloride diffusion coefficients among its components. RCA sourced from parent concrete may contain residual chloride ions. Given the diverse sources of RCA, the initial chloride ions inside aggregates were not incorporated into the model. The chloride diffusion coefficient of new mortar can be calculated as follows [30]:

(6) $$D_{nm}=\frac{2{\emptyset_{p}}^{2.75}D_{t}}{{\emptyset_{p}}^{1.75}\left(3-\emptyset_{p}\right)+14.44{\left(1-\emptyset_{p}\right)}^{2.75}}$$

where $D_{t}$ is the chloride diffusion coefficient in the pore solution, and $D_{t}$ = $1.07\times 10^{-10}\;m^{2}/s$, $\emptyset_{p}$ is the new mortar porosity, and $\emptyset_{p}$ is determined as follows [31]:

(7) $$\emptyset_{p}=\frac{w/b-0.17\gamma }{w/b+0.32}$$

where w/b is the water-binder ratio, γ is the hydration level, with a scale from 0 to 1.

In reference [11], it was mentioned that the chloride diffusion coefficient of old mortar was 0.2 to 5 times higher than that of new mortar. Additionally, the chloride diffusion coefficient of ITZ was reported to be 1.3 to 16.2 times that of mortar. In this study, the chloride diffusion coefficient of old mortar was two times higher than that of new mortar, and the chloride diffusion coefficient of ITZ was ten times higher than that of mortar, which is consistent with the values adopted in references [15,17]. By contrast, original aggregates have an extremely low chloride diffusion coefficient, which is set to 0 m^2^/s in this study. As a result, the chloride concentration inside original aggregates remains zero, and this assumption may slightly affect chloride diffusion pathways.

## 3. Experimental Program

### 3.1. Materials and Mix Proportions

This study adopted P·O 42.5 cement. Fly ash was supplied from a local concrete company and meets Grade I requirements specified in GB/T 1596-2017 standard [32]. Superfine fly ash was prepared by grinding fly ash in a ball mill. The chemical compositions of cement and fly ash are shown in Table 1. Natural coarse aggregates (NCA) consisted of gravel with diameters ranging from 5 mm to 20 mm. RCA was sourced from C&DW and had the same diameter range, with an old mortar content of 44.8%. The physical properties of coarse aggregates are shown in Table 2. Fine aggregates were obtained from river sand with an apparent density of 2620 kg/m^3^ and a fineness modulus of 2.8. The water-binder ratio was 0.4. The mix proportions of RAC are shown in Table 3.

### 3.2. Salt Freeze-Thaw

**Figure 2:**
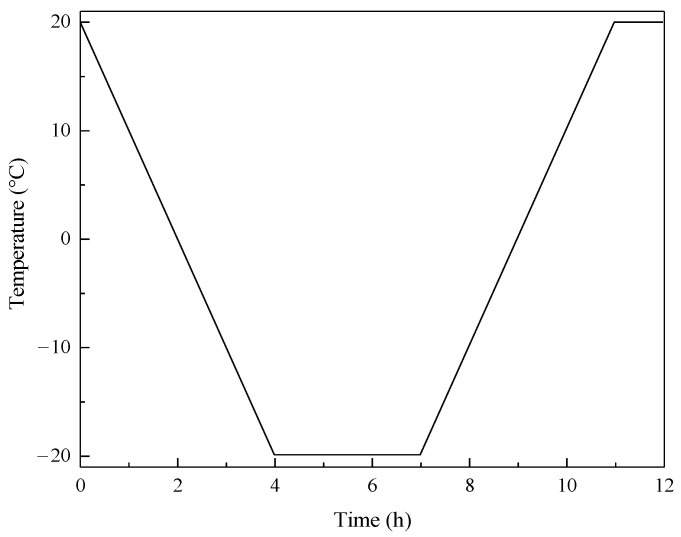
Freeze-thaw process.

The single-side salt freeze-thaw method was adopted in accordance with GB/T 50082-2009 [33]. The RAC specimens, with dimensions of 100 mm × 100 mm × 100 mm, underwent a 24-day curing process in an environment maintained at 20±2 °C and 95% relative humidity. To ensure one-dimensional chloride penetration, the specimens were coated with epoxy resin on four surfaces and then immersed in water for four days. Subsequently, the specimens were subjected to freeze-thaw cycles in a box with 3% sodium chloride solution. The test was terminated after 120 freeze-thaw cycles, with a total test duration of 60 days. The freeze-thaw process is shown in Figure 2.

### 3.3. Compressive Strength

After 120 freeze-thaw cycles, the compressive strength of the specimens was measured using a universal testing machine, with the loading rate controlled at 0.5 MPa/s. The final compressive strength value was taken as the arithmetic average of test results from three parallel specimens.

### 3.4. Relative Dynamic Elastic Modulus

The relative dynamic elastic modulus *E_f_* is calculated by the following equation:

(8) $$E_{f}=\frac{f_{n}^{2}}{f_{0}^{2}}\times 100$$

where *f*_0_ is the transverse fundamental frequency of the specimen before freeze-thaw cycles (Hz), and *f_n_* is the transverse fundamental frequency of the specimen after n freeze-thaw cycles (Hz).

### 3.5. Mass Loss

The mass loss *M_n_* is calculated by the following equation:

(9) $$M_{n}=\frac{G_{0}-G_{n}}{G_{0}}\times 100$$

where *G*_0_ is the mass of the saturated specimen (g), and *G_n_* is the mass of the specimen subjected to n freeze-thaw cycles.

### 3.6. Chloride Content

Specimens were taken out of the freeze-thaw testing machine, and successive 5 mm-thick layers were precisely chipped off from the erosion surface before being ground into fine powder with an agate mortar and pestle. The powder was sieved through a 0.16 mm sieve for subsequent testing. The test procedures followed JGJ/T 322-2013 [34], and the chloride content in RAC was calculated by using Equation (10):

(10) $$P_{h}=\frac{C_{AgNO_{3}}\times V_{1}\times V_{2}\times 0.03545}{m_{0}\times V_{3}}\times 100\%$$

where $P_{h}$ is the chloride content in RAC (%), $C_{{AgNO}_{3}}$ is the concentration of silver nitrate solution (mol/L), $m_{0}$ is the sample powder mass (g), $V_{1}$ is the consumption of silver nitrate solution (mL), $V_{2}$ is the volume of distilled water (mL), $V_{3}$ is the filtrate volume (mL).

## 4. Results

### 4.1. Compressive Strength

Table 4 shows the compressive strength results of all specimens after 120 freeze-thaw cycles. FA0R0 exhibits the highest compressive strength of 46.7 MPa. At a 15% dosage of fly ash or superfine fly ash, the compressive strength values of FA15R0 and SF15R0 decrease to 41.9 MPa and 39.5 MPa. At a 25% RCA substitution ratio, FA0R25 yields a compressive strength of 37.8 MPa. The compressive strength of FA15R25 increases to 42.6 MPa, whereas that of SF15R25 reaches 38.4 MPa. When the fly ash or superfine fly ash dosage is elevated to 30%, the compressive strength values of FA30R25 and SF30R25 drop to 29.1 MPa and 28.2 MPa. Among all specimens, FA15R50 exhibits the lowest compressive strength at merely 26.8 MPa. These observed strength variation trends clearly reflect the influences of RCA substitution ratio and fly ash/superfine fly ash dosage on the compressive strength of specimens after freeze-thaw cycles.

### 4.2. Relative Dynamic Elastic Modulus

Table 5 shows the relative dynamic elastic modulus results for all specimens subjected to 30, 60, 90, and 120 freeze-thaw cycles. For all specimens, the relative dynamic elastic modulus exhibits a gradual downward trend as the number of freeze-thaw cycles increases. After 120 freeze-thaw cycles, the corresponding values fall within the range of 53% to 66%. Specifically, the relative dynamic elastic modulus of FA0R0 drops to 66%, whereas that of all other specimens is lower. FA30R25, SF30R25 and FA15R50 consistently show relatively lower values. Overall, the variation pattern of relative dynamic elastic modulus across all specimens after 120 freeze-thaw cycles is broadly consistent with the trend of compressive strength.

### 4.3. Mass Loss

Table 6 shows the mass loss of all specimens after 30, 60, 90 and 120 freeze-thaw cycles. As the number of freeze-thaw cycles increases, the mass loss of all specimens gradually shifts from negative to positive values, indicating that the specimen mass generally follows a trend of initial increase followed by decrease. After 120 freeze-thaw cycles, FA30R25, SF30R25 and FA15R50 exhibit relatively higher mass loss, which is generally consistent with the variation trend of the relative dynamic elastic modulus.

## 5. Validation and Discussion

**Figure 3:**
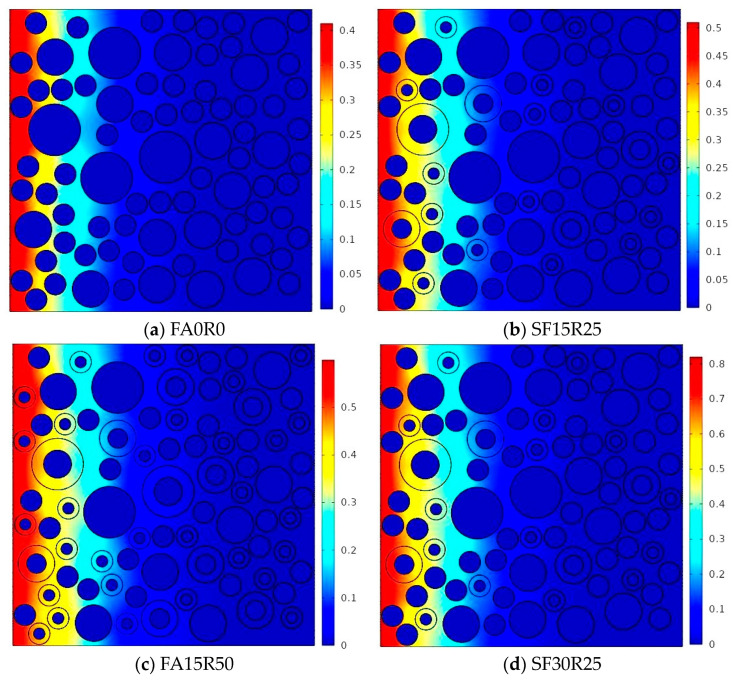
Chloride concentration distribution at RCA substitution ratios ranging from 0–50%.

The simulated values were averaged across three locations at the same depths. Freeze-thaw cycles can significantly accelerate the process of chloride penetration process within concrete. According to Fick’s second law, the chloride concentration of concrete is determined by the chloride diffusion coefficient and diffusion time. Freeze-thaw exposure increases the chloride diffusion coefficient of each phase in concrete to varying degrees; therefore, the chloride penetration effect under freeze-thaw conditions can be equivalently regarded as the result of natural diffusion over a longer duration. Upon completing 120 freeze-thaw cycle tests, a comparative analysis of the measured chloride data against COMSOL simulation results was performed. The comparison indicates that the penetration effect observed in the test corresponds to approximately 800 days of penetration in the mesoscale model. After repeated comparisons, it is finally confirmed that the highest degree of agreement between simulated and experimental values is achieved when the penetration time is set to 780 days. The numerical parameters of the RAC model are shown in Table 7. Figure 3 shows the chloride concentration distribution in RAC at RCA substitution ratios ranging from 0–50%.

### 5.1. Effect of RCA Substitution Ratios on Chloride Concentration

**Figure 4:**
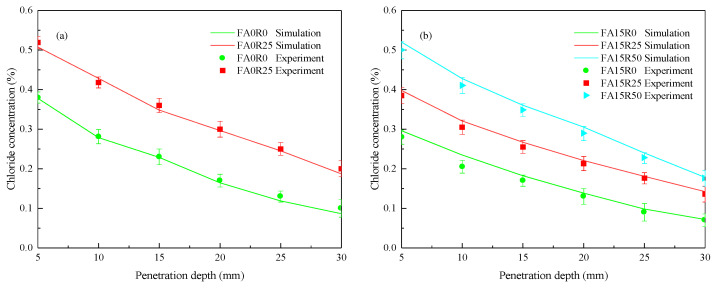
Comparison of chloride concentration between experimental data and simulated values at different RCA substitution ratios: (**a**) FA0R0 and FA0R25; (**b**) FA15R0, FA15R25 and FA15R50.

Figure 4 shows the comparison of chloride concentration between experimental data and simulated values at different RCA substitution ratios. The error bars in all figures represent the standard deviation of triplicate test results (*n* = 3). The experimental data for FA0R0 and FA0R25 closely align with the simulated values. However, the experimental data for FA15R0, FA15R25, and FA15R50 are lower than the simulated values. Adding 15% fly ash to RAC enhances its resistance to chloride penetration. This enhancement is attributed to the pozzolanic reaction of fly ash, which facilitates the formation of hydrated calcium silicate (C-S-H) gel. Consequently, the concrete becomes denser, leading to a reduction in chloride concentration. This elucidates why the experimental data for FA15R0, FA15R25, and FA15R50 are lower than the simulated values.

In Figure 4a, at a depth of 5 mm, the chloride concentration from experimental data reaches 0.38% for FA0R0, while the experimental data of FA0R25 shows a chloride concentration of 0.52%. In Figure 4b, at a depth of 10 mm, the experimental data of FA15R50 exhibits an 82.61% increase in chloride concentration compared to the experimental data of FA15R0. Similarly, the experimental data of FA15R25 demonstrate a 34.78% increase in chloride concentration relative to the experimental data of FA15R0. Increasing the RCA substitution ratio reduces the resistance of RAC to chloride penetration during freeze-thaw cycles. The rising content of adhesive old mortar is the primary reason for this phenomenon, leading to increased porosity and microcracks in RAC. Freeze-thaw cycles generate new cracks and pores, providing more pathways for chloride penetration.

### 5.2. Effect of Fly Ash and Superfine Fly Ash Dosages on Chloride Concentration

**Figure 5:**
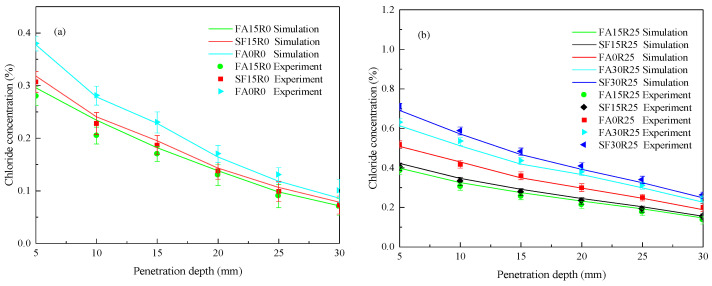
Comparison of chloride concentration between experimental data and simulated values at varying dosages of fly ash and superfine fly ash: (**a**) FA15R0, SF15R0 and FA0R0; (**b**) FA15R25, SF15R25, FA0R25, FA30R25 and SF30R25.

Figure 5 shows the comparison of chloride concentration between experimental data and simulated values at varying dosages of fly ash and superfine fly ash. The experimental data for FA30R25 and SF30R25 exceed the simulated values. Adding 30% fly ash to RAC reduces its resistance to chloride penetration. Excessive fly ash impedes the hydration reaction, increasing the concrete’s chloride concentration. Therefore, the experimental data for FA30R25 exceed the simulated values. In Figure 5a, the experimental chloride concentration values for FA0R0 are the highest, while those for FA15R0 show the largest drop. At a depth of 15 mm, the experimental chloride concentration reaches 0.23% for FA0R0, 0.2% for SF15R0, and 0.18% for FA15R0. In Figure 5b, FA15R25 presents the most significant reduction in experimental chloride concentration values compared with those of FA0R25, followed by those of SF15R25. SF30R25 exhibits the largest rise in experimental chloride concentration values compared with those of FA0R25, followed by those of FA30R25.

## 6. Simulation and Discussion

The RCA volume fraction, ITZ thickness, and adhesive ratio of old mortar were further analyzed quantitatively. It was assumed that the new ITZ thickness was equal to that of the old ITZ. The chloride concentration at the model’s boundary was set at 1.5%. The chloride diffusion coefficients of the model are shown in Table 8.

### 6.1. RCA Volume Fraction

**Figure 6:**
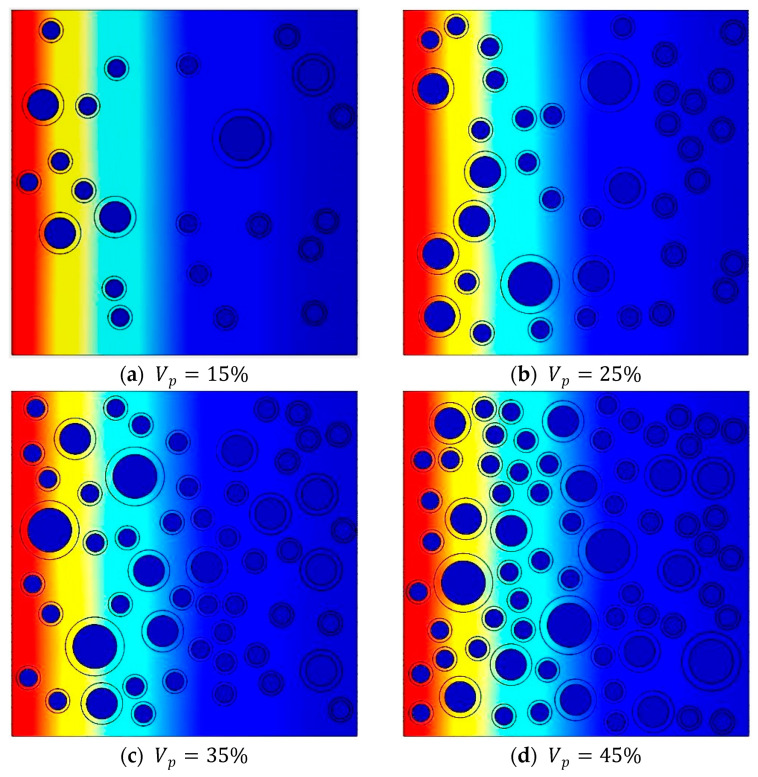
Chloride concentration distribution at different RCA volume fractions.

**Figure 7:**
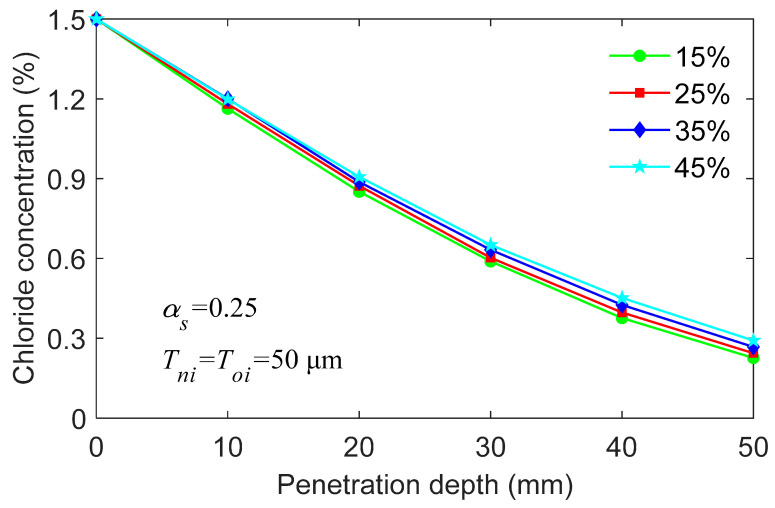
Effect of different RCA volume fractions on chloride concentration.

The chloride concentration distribution in RAC at different RCA volume fractions ($V_{p}=$ 15%, 25%, 35%, and 45%) after 1200 days was calculated, as shown in Figure 6. The adhesive ratio of old mortar was 0.25. Both the new and old ITZ had a thickness of 50 μm. Figure 7 shows the effect of different RCA volume fractions on chloride concentration. The chloride concentration values increase with a higher RCA volume fraction. At a depth of 50 mm, the chloride concentration values are 0.23%, 0.24%, 0.27%, and 0.29%, respectively. As the RCA volume fraction increases, there is a corresponding rise in weak regions such as adhesive old mortar. Consequently, the resistance of RAC to chloride penetration decreases.

**Figure 8:**
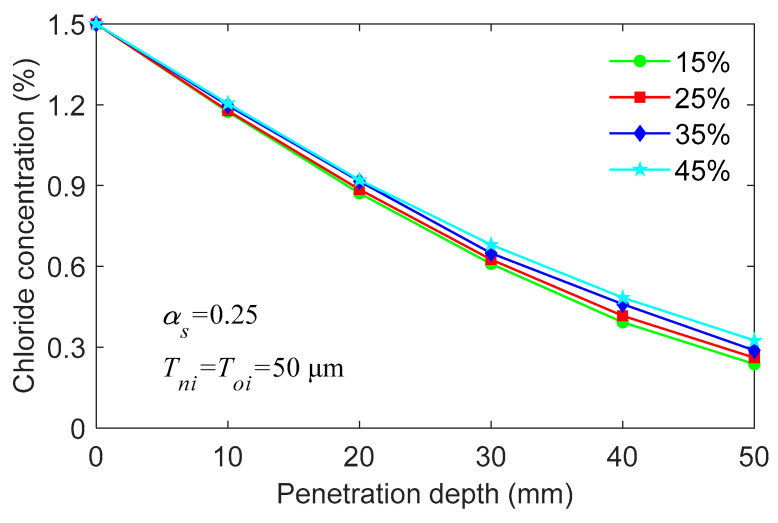
Chloride concentration distribution when the chloride diffusion coefficient of old mortar is 2.5 times that of new mortar.

A parametric sensitivity analysis is carried out in this section to verify the reliability of the main conclusions of this paper. First, the chloride diffusion coefficient of old mortar is adjusted to 2.5 times that of new mortar, while all other parameters remain unchanged. The resulting chloride concentration distribution is shown in Figure 8. At a depth of 50 mm, the chloride concentration values are 0.24%, 0.26%, 0.29%, and 0.32%, respectively.

**Figure 9:**
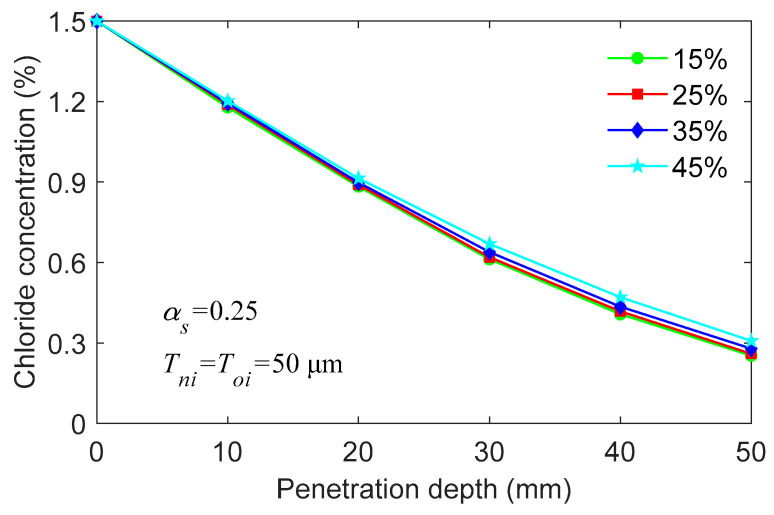
Chloride concentration distribution when the chloride diffusion coefficient of ITZ is 13 times that of mortar.

Then, the chloride diffusion coefficient of ITZ is adjusted to 13 times that of mortar, with all other parameters kept the same. The chloride concentration distribution is shown in Figure 9. At a depth of 50 mm, the chloride concentrations are 0.25%, 0.26%, 0.28%, and 0.31%, respectively. Overall, the core conclusion that the chloride penetration resistance of RAC decreases with increasing RCA volume fraction remains unchanged. This demonstrates that the parameter values adopted in this paper are reasonable and the research conclusions are reliable.

### 6.2. ITZ Thickness

**Figure 10:**
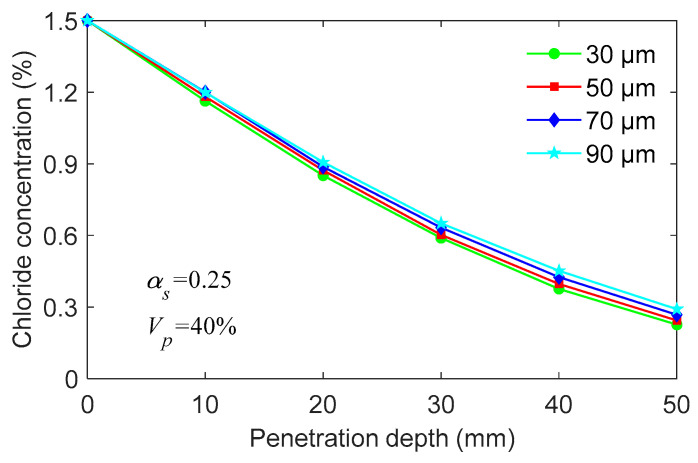
Effect of different ITZ thicknesses on chloride concentration.

While the ITZ thickness is relatively small, its effect on chloride concentration in RAC should not be underestimated. Figure 10 shows the effect of different ITZ thicknesses ($T_{ni}=T_{oi}=$ 30 μm, 50 μm, 70 μm, and 90 μm) on chloride concentration. As the ITZ thickness increases, there is a consistent rise in chloride concentration. At a depth of 40 mm, the chloride concentration values are 0.38%, 0.40%, 0.43%, and 0.45%, respectively. Increasing the ITZ thickness reduces the resistance of RAC to chloride penetration.

### 6.3. Adhesive Ratio of Old Mortar

**Figure 11:**
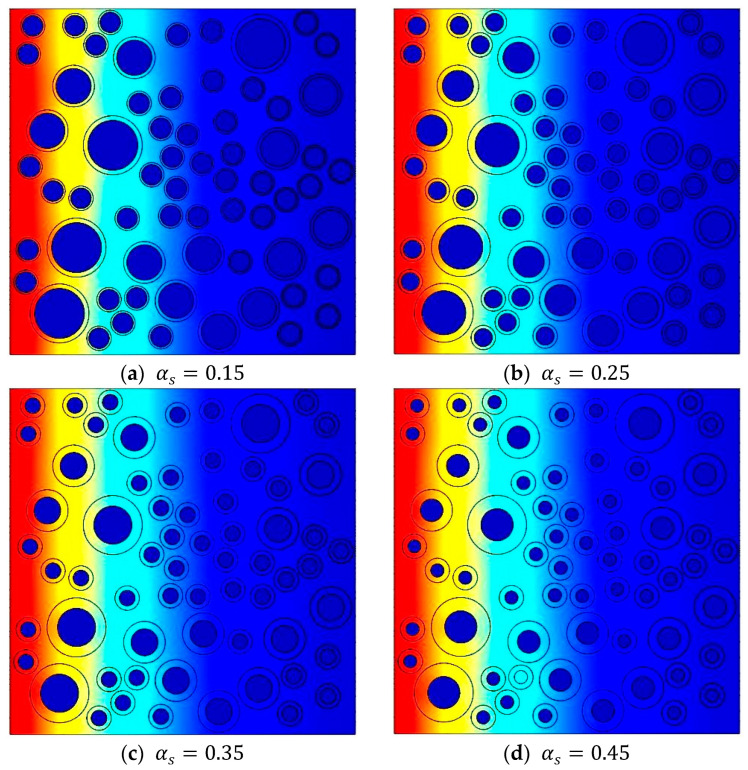
Chloride concentration distribution at different adhesive ratio of old mortar.

**Figure 12:**
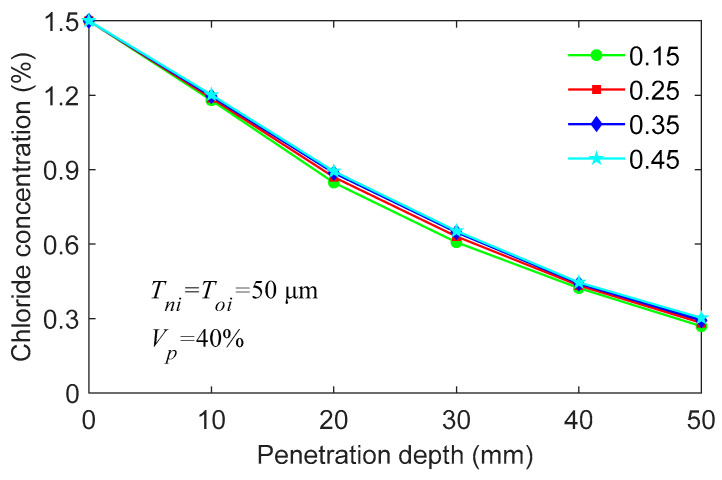
Effect of different adhesive ratios of old mortar on chloride concentration.

The chloride concentration distribution in RAC at different adhesive ratios of old mortar ($\alpha_{s}=$ 0.15, 0.25, 0.35, and 0.45) was extracted, as shown in Figure 11. Figure 12 shows the effect of different adhesive ratios of old mortar on chloride concentration. The chloride concentration values increase as the adhesive ratio of old mortar rises. Increasing the adhesive rate of old mortar reduces the resistance of RAC to chloride penetration. At a depth of 30 mm, the chloride concentration values are 0.61%, 0.63%, 0.65%, and 0.66%, respectively. Reducing the adhesive ratio of old mortar is recommended to enhance the resistance of RAC to chloride penetration.

### 6.4. Engineering Application Suggestions for RCA

The utilization of RCA in practical engineering remarkably reduces the demand for natural aggregates, facilitates the recycling of C&DW, curbs carbon emissions, and mitigates the environmental pressure arising from C&DW landfilling, which contributes to ecological conservation. The quality of RCA can be assessed via critical indices including water absorption and crushing value. As a general rule, RCA with lower water absorption exhibits superior overall performance. Two categories of physical and chemical pretreatment approaches are available to strip old mortar from RCA surfaces for performance enhancement. Physical pretreatments cover mechanical grinding and microwave thermal treatment, while chemical modification involves immersing RCA in conditioning agents such as acidic solutions and water glass solutions. From the perspective of long-term structural service reliability, the RCA substitution ratio is suggested to be limited to 50% for on-site applications. Such a dosage threshold can effectively avoid severe degradation in concrete mechanical performance and lower potential durability hazards originating from inconsistent RCA quality.

## 7. Conclusions

In this study, the damage degree of RAC under salt freeze-thaw environments was characterized through the indices of compressive strength, relative dynamic elastic modulus, and mass loss. A five-phase mesoscale model of RAC was established. This study innovatively combines numerical simulation with experimental validation to investigate the effects of different RCA substitution ratios, as well as varying dosages of fly ash and superfine fly ash, on chloride concentration in RAC during freeze-thaw cycles. The main conclusions are drawn as follows:

(1) Increasing the RCA substitution ratio significantly reduces the resistance of RAC to chloride penetration. This phenomenon is primarily attributed to increased old mortar, which contains more microcracks. Under freeze-thaw cycles, these defects are further amplified, thus forming transport pathways for chloride penetration.
(2) Adding 15% fly ash to RAC enhances its resistance to chloride penetration, whereas adding 30% fly ash exerts a detrimental effect.
(3) Controlling the RCA volume fraction, ITZ thickness, and adhesive ratio of old mortar at lower levels can effectively improve the resistance of RAC to chloride penetration.

Overall, this study reveals the multi-factor mechanism of chloride penetration in RAC under freeze-thaw environments through the combination of numerical simulation and experimental validation. The findings provide a quantitative theoretical basis and practical guidance for the durability design of RAC in cold regions. This study mainly explores the effects of RCA substitution ratios, fly ash, and superfine fly ash dosages on chloride concentration, with limited research variables. Future research will further improve the chloride penetration mechanism of RAC under salt freeze-thaw environments by considering more influencing factors, including freeze-thaw cycle times, chloride solution concentration, crack width, and other supplementary cementitious materials.

## Figures and Tables

**Table 1:** Chemical compositions of cement and fly ash (%).

Composition	SiO_2_	Al_2_O_3_	CaO	MgO	SO_3_	Fe_2_O_3_	Na_2_O	Loss
Cement	21.35	5.19	62.17	2.18	2.65	3.48	0.23	1.47
Fly ash	51.76	29.31	5.68	0.84	1.52	4.93	0.21	2.91

**Table 2:** Physical properties of coarse aggregates.

	Particle Size (mm)	Apparent Density (kg/m^3^)	Water Absorption (%)	Crushing Value (%)
NCA	5–20	2670	0.5	7.9
RCA	5–20	2580	4.8	16.3

**Table 3:** Mix proportions of RCA (kg/m^3^).

	Water	Cement	Fly Ash	Superfine Fly Ash	Sand	NCA	RCA
FA0R0	175	438	0	0	572	1216	0
FA15R0	175	372	66	0	572	1216	0
SF15R0	175	372	0	66	572	1216	0
FA0R25	175	438	0	0	572	912	304
FA15R25	175	372	66	0	572	912	304
FA30R25	175	306	132	0	572	912	304
SF15R25	175	372	0	66	572	912	304
SF30R25	175	306	0	132	572	912	304
FA15R50	175	372	66	0	572	608	608

FAx: The substitution level of cement by fly ash. SFx: The substitution level of cement by superfine fly ash. Rx: The substitution level of NCA by RCA.

**Table 4:** Compressive strength of all specimens after 120 freeze-thaw cycles.

	FA0R0	FA15R0	SF15R0	FA0R25	FA15R25	FA30R25	SF15R25	SF30R25	FA15R50
Compressive strength (MPa)	46.7	41.9	39.5	37.8	42.6	29.1	38.4	28.2	26.8

**Table 5:** Relative dynamic elastic modulus results for all specimens.

	FA0R0	FA15R0	SF15R0	FA0R25	FA15R25	FA30R25	SF15R25	SF30R25	FA15R50
0	100%	100%	100%	100%	100%	100%	100%	100%	100%
30	99%	98%	98%	97%	99%	94%	96%	93%	92%
60	91%	89%	87%	88%	89%	82%	86%	81%	83%
90	79%	76%	75%	73%	74%	68%	72%	70%	69%
120	66%	64%	62%	61%	60%	54%	59%	53%	55%

**Table 6:** Mass loss of all specimens.

	FA0R0	FA15R0	SF15R0	FA0R25	FA15R25	FA30R25	SF15R25	SF30R25	FA15R50
30	−0.9%	−0.7	−1.0%	−1.2%	−0.8%	−1.3%	−0.6%	−1.1%	−1.4%
60	−0.2%	−0.3%	−0.1%	0.2%	0.1%	0.4%	0.3%	0.6%	0.7%
90	0.6%	0.8%	0.9%	1.1%	1.2%	2.0%	1.3%	2.2%	1.9%
120	1.8%	2.1%	2.3%	2.5%	2.6%	3.5%	2.8%	3.6%	3.4%

**Table 7:** Numerical parameters of the RAC model.

Symbol	Parameter	Value
$T_{d}$	Diffusion time	780 days
$V_{p}$	Volume fraction of coarse aggregate	47%
$\alpha_{s}$	Adhesive rate of old mortar	0.45
w/b	Water-binder ratio	0.4
$D_{nm}$	Chloride diffusion coefficient of new mortar	$3.8553\times 10^{-12}\;m^{2}/s$
$D_{om}$	Chloride diffusion coefficient of old mortar	$7.7106\times 10^{-12}\;m^{2}/s$
$D_{ni}$	Chloride diffusion coefficient of the new ITZ	$3.8553\times 10^{-11}\;m^{2}/s$
$D_{oi}$	Chloride diffusion coefficient of the old ITZ	$7.7106\times 10^{-11}\;m^{2}/s$
$D_{agg}$	Chloride diffusion coefficient of original aggregate	$0\;m^{2}/s$
$T_{ni}$	Thickness of the new ITZ	100 μm
$T_{oi}$	Thickness of the old ITZ	100 μm

**Table 8:** Chloride diffusion coefficients of the model.

Symbol	Parameter	Value
$D_{nm}$	Chloride diffusion coefficient of new mortar	$5.4492\times 10^{-12}\;m^{2}/s$
$D_{om}$	Chloride diffusion coefficient of old mortar	$1.0898\times 10^{-11}\;m^{2}/s$
$D_{ni}$	Chloride diffusion coefficient of the new ITZ	$5.4492\times 10^{-11}\;m^{2}/s$
$D_{oi}$	Chloride diffusion coefficient of the old ITZ	$1.0898\times 10^{-10}\;m^{2}/s$
$D_{agg}$	Chloride diffusion coefficient of original aggregate	$0\;m^{2}/s$

## Data Availability

The original contributions presented in this study are included in the article. Further inquiries can be directed to the corresponding authors.

## References

[1] Pan C., Song Y., Wang J., Zhan S., Unluer C., Ruan S. (2023). Unlocking the role of recycled aggregates in the performance enhancement and CO_2_ capture of reactive magnesia cement formulations. Cem. Concr. Res..

[2] Song Y., Wang J., Huang Y., Wang J., Weng Y., Ma R., Pang K., Ruan S. (2025). Effects of varying grades/pretreatments of recycled aggregates on the development of pore structures and ITZs within reactive magnesia cement (RMC) concrete. Cem. Concr. Res..

[3] Jin L., Dong T., Fan T., Duan J., Yu H., Jiao P., Zhang W. (2022). Prediction of the chloride diffusivity of recycled aggregate concrete using artificial neural network. Mater. Today Commun..

[4] Song Y., Diao X., Xu R., Wang X., Han Y., Shi T., Fang B., Liu Y., Corr D.J. (2025). A comprehensive review of mineral carbonation of civil engineering materials: A bibliometric analysis. Environ. Sci. Technol..

[5] Yuan W., Mao L., Li L. (2023). A two-step approach for calculating chloride diffusion coefficient in concrete with both natural and recycled concrete aggregates. Sci. Total Environ..

[6] Bahraq A., Jose J., Shameem M., Maslehuddin M. (2022). A review on treatment techniques to improve the durability of recycled aggregate concrete: Enhancement mechanisms, performance and cost analysis. J. Build. Eng..

[7] Gao S., Guo J., Gong Y., Ban S., Liu A. (2022). Study on the penetration and diffusion of chloride ions in interface transition zone of recycled concrete prepared by modified recycled coarse aggregates. Case Stud. Constr. Mater..

[8] Liu Q., Feng G., Xia J., Yang J., Li L. (2018). Ionic transport features in concrete composites containing various shaped aggregates: A numerical study. Compos. Struct..

[9] Ji Y., Wang Z., Inose S., Tsuchiya S., Yamamoto T., Ishida T., Maekawa K. (2025). Numerical study of macro-cell corrosion on reinforced concrete induced by chloride attack. Cem. Concr. Compos..

[10] Li Q., Zhang W., Shao W., Shi D. (2024). Numerical modeling of non-uniform corrosion of concrete reinforcement considering calcium leaching and chloride diffusion coupling effect. Corros. Sci..

[11] Xiao J., Ying J., Shen L. (2012). FEM simulation of chloride diffusion in modeled recycled aggregate concrete. Constr. Build. Mater..

[12] Ying J., Xiao J., Shen L., Bradford M. (2013). Five-phase composite sphere model for chloride diffusivity prediction of recycled aggregate concrete. Mag. Concr. Res..

[13] Srubar W. (2015). Stochastic service-life modeling of chloride-induced corrosion in recycled-aggregate concrete. Cem. Concr. Compos..

[14] Liu Q., Easterbrook D., Yang J., Li L. (2015). A three-phase, multi-component ionic transport model for simulation of chloride penetration in concrete. Eng. Struct..

[15] Hu Z., Mao L., Xia J., Liu J., Gao J., Yang J., Liu Q. (2018). Five-phase modelling for effective diffusion coefficient of chlorides in recycled concrete. Mag. Concr. Res..

[16] Yu Y., Lin L. (2020). Modeling and predicting chloride diffusion in recycled aggregate concrete. Constr. Build. Mater..

[17] Wu T., Jin L., Wang Z., Qiao L., Zhou P. (2026). Transport behavior of sulfate ions in concrete subjected to chloride ions: A mesoscale and multiphase model. J. Mater. Civ. Eng..

[18] Yao M., Lian H., Chai S., Shang H., Feng T. (2026). The Effect of polyaluminum chloride (PAC) residue on the mechanical properties and freeze-thaw resistance of cement mortar. Civ. Environ. Eng..

[19] Yu Y., Wang J., Wang N., Wu C., Zhang X., Wang D., Ma Z. (2021). Combined freeze-thaw and chloride attack resistance of concrete made with recycled brick-concrete aggregate. Materials.

[20] Tang H., Yang Y., Li H., Xiao L., Ge Y. (2023). Effects of chloride salt erosion and freeze-thaw cycle on interface shear behavior between ordinary concrete and self-compacting concrete. Structures.

[21] Wang F., Qin X., Pang W., Wang W. (2021). Performance deterioration of asphalt mixture under chloride salt erosion. Materials.

[22] Dong X., Yu T., Zhang Q., Bui T. (2023). Multiscale freezing-thaw in concrete: A numerical study. Compos. Struct..

[23] Kuosa H., Ferreira R., Holt E., Leivo M., Vesikari E. (2014). Effect of coupled deterioration by freeze-thaw, carbonation and chlorides on concrete service life. Cem. Concr. Compos..

[24] Jiang W., Shen X., Xia J., Mao L., Yang J., Liu Q. (2018). A numerical study on chloride diffusion in freeze-thaw affected concrete. Constr. Build. Mater..

[25] Xue Q., Zheng T.-Y., Wang J., Zhang J.-J., Xia W., Cui S.-A. (2025). Study on chloride permeability and chloride ion transport of fiber-reinforced cementitious composite repair system. Buildings.

[26] Yin S., Jing L., Yin M., Wang B. (2019). Mechanical properties of textile reinforced concrete under chloride wet-dry and freeze-thaw cycle environments. Cem. Concr. Compos..

[27] Gao P., Gao X., Tong L., Meng Z., Xiong Q., Ferrara L., Liu Q. (2026). Multi-physics coupling model of chloride-induced rebar corrosion and concrete cracking in reinforced concrete structure. Comput. Struct..

[28] Xue W., Twenda C., Alam M.S., Xu L., Wang Z. (2023). Experimental study on seepage characteristics and stress sensitivity of desulfurization gypsum based concrete under triaxial stress. J. Mater. Res. Technol..

[29] Walraven J., Reinhardt H. (1981). Theory and Experiments on the Mechanical Behaviour of Cracks in Plain and Reinforced Concrete Subjected to Shear Loading. HERON.

[30] Zheng J., Zhou X. (2008). Analytical Solution for the Chloride Diffusivity of Hardened Cement Paste. J. Mater. Civ. Eng..

[31] Sun G., Zhang Y., Sun W., Liu Z., Wang C. (2011). Multi-scale prediction of the effective chloride diffusion coefficient of concrete. Constr. Build. Mater..

[32] (2017). Fly Ash Used for Cement and Concrete.

[33] (2009). Standard for Test Methods of Long-Term Performance and Durability of Ordinary Concrete.

[34] (2013). Technical Specification for Test of Chloride Ion Content in Concrete.

